# Clinical significance of preoperative serum vascular endothelial growth factor, interleukin-6, and C-reactive protein level in colorectal cancer

**DOI:** 10.1186/1471-2407-10-203

**Published:** 2010-05-14

**Authors:** Kyung A Kwon, Sung Hyun Kim, Sung Yong Oh, Suee Lee, Jin-Yeong Han, Kyeong Hee Kim, Ri Young Goh, Hong Jo Choi, Ki Jae Park, Mee Sook Roh, Hyo-Jin Kim, Hyuk-Chan Kwon, Jong Hoon Lee

**Affiliations:** 1 Department of Internal Medicine, Dong-A university College of Medicine, Busan, Korea; 2 Department of Laboratory Medicine, Dong-A university College of Medicine, Busan, Korea; 3 Department of Surgery, Dong-A university College of Medicine, Busan, Korea; 4 Department of Pathology, Dong-A university College of Medicine, Busan, Korea; 5 Medical Research Center for Cancer Molecular Therapy, Dong-A University College of Medicine, Busan, Korea

## Abstract

**Background:**

Angiogenesis is a multistep process in which many growth factors and cytokines have an essential role. Vascular endothelial growth factor (VEGF) is a potent angiogenic agent that acts as a specific mitogen for vascular endothelial cells through specific cell surface receptors. The interleukin-6 (IL-6) pathway is another mechanism linking angiogenesis to malignancy. C-reactive protein (CRP), a representative marker for inflammation, is known for its association with disease progression in many cancer types. The aim of this study was to determine preoperative serum levels of VEGF, IL-6, and CRP in colorectal carcinoma, and to correlate them with disease status and prognosis.

**Methods:**

A 132 of 143 patients who underwent curative resection for colorectal cancer were enrolled in this study. 11 patients with resection margin positive were excluded. Factors considered in analysis of the relationship between VEGF, IL-6, and CRP and histological findings. Patient prognosis was investigated. Serum levels of VEGF and IL-6 were assessed using Enzyme-Linked Immuno-Sorbent Assay (ELISA), and CRP was measured using immunoturbidimetry.

**Results:**

Median follow-up duration was 18.53 months (range 0.73-43.17 months) and median age of the patients was 62 years (range, 26-83 years). Mean and median levels of VEGF and CRP in colorectal cancer were significantly higher than in the normal control group; 608 vs. 334 pg/mL and 528 (range 122-3242) vs. 312 (range 16-1121) (*p *< 0.001); 1.05 mg/dL vs. 0.43 mg/dL and 0.22 (range 0.00-18.40) vs. 0.07 (range 0.02-6.94) (*p *= 0.002), respectively. However mean and median level of IL-6 in patients were not significantly higher than in control; 14.33 pg/mL vs. 5.65 pg/mL and 6.00 (range 1.02-139.17) vs. 5.30 (4.50-13.78) (*p *= 0.327). Although IL-6 and CRP levels were not correlated with other pathological findings, VEGF level was significantly correlated with tumor size (*p *= 0.012) and CEA (*p *= 0.038). When we established the cutoff value for VEGF (825 pg/mL), IL-6 (8.09 pg/mL), and CRP (0.51 mg/dL) by Receiver Operating Characteristic (ROC) curve, we noted that high VEGF levels tended to reduce overall survival (*p *= 0.053), but not significantly. However, IL-6 and CRP demonstrated no significance with regard to disease free survival (*p *= 0.531, *p *= 0.701, respectively) and overall survival (*p *= 0.563, *p *= 0.572, respectively). Multivariate analysis showed that VEGF (*p *= 0.032), CEA (*p *= 0.012), lymph node metastasis (*p *= 0.002), and TNM stage (*p *= 0.025) were independently associated with overall survival.

**Conclusions:**

Preoperative serum VEGF and CRP level increased in colorectal cancer patients. High VEGF level has been proposed as a poor prognostic factor for overall survival in patients with colorectal cancer.

## Background

Colorectal cancer is a common malignant disease, accounting for approximately 15% of all human cancers [1]. In Korea, it is the third most common cancer and the fourth cause of cancer death and its incidence is increasing [2]. Incidence of colorectal cancer has grown rapidly since 1990, and is influenced by eating and lifestyle habits that include increased meat intake and reduced fiber intake, alcohol consumption, and smoking. Current diagnostic techniques and universal colonoscopy have enabled relatively early diagnosis of colorectal cancer, and the cure rate for early stage colorectal cancer is high, at up to 80-90% [3]. Although AJCC TNM classification is useful for staging of colorectal cancer patients and selection for specific treatment, it is not sufficient, as outcome may vary in different patients at the same stage, indicating that conventional staging procedures may not provide accurate prediction of cancer prognosis. Independent of the TNM Stage, the carcinoembryonic antigen (CEA) is used for prediction of prognosis; however, diagnostic sensitivity of CEA is unsatisfactory. Therefore, an appropriate molecular marker is a necessity for adequate treatment of aggressive CRC patients with adjuvant systemic chemotherapy or targeted therapy.

Because new blood vessel formation provides a route for spread of tumor cells to distant organs, angiogenesis as a central process in progression of solid tumors is a well-established aspect of cancer biology [4]. The process may result from an imbalance between positive and negative angiogenic regulators released by both tumor cells and host cells [5]. Tumor angiogenesis and its promoting growth factors have been associated with advanced stage, lymph node involvement and poor survival in a variety of human cancers [6-8]. One of the major pathways associated with this process is that of the vascular endothelial growth factor (VEGF) family of proteins and receptors. Indeed, many tumor cell lines secrete VEGF in vitro, and high VEGF mRNA levels can be detected by in situ hybridization in most human tumors, including lung, breast, and gastrointestinal carcinoma [9]. Both VEFG and its receptor are expressed at high levels in metastatic human colon carcinomas and in tumor associated endothelial cells, and production of these two proteins correlates directly with degree of tumor vascularization [10].

Interleukin-6 (IL-6) is a multipoietic cytokine produced by many cell types. IL-6 acts on a wide range of tissues and cell lines, and induces cell growth and differentiation, production and expression of other cytokines, and acute-phase protein synthesis. IL-6 also promotes growth arrest [11,12], and promotes angiogenesis through induction of VEGF expression [13,14].

C-reactive protein (CRP) is an acute reactant protein that is increased under conditions of infection, trauma, tissue necrosis, tumor, and several types of inflammatory disease. It reflects inflammatory status and is a component of the inflammatory response of the immune system [15,16]. Several studies have reported that in occurrence, progression, metastasis, and recurrence of colorectal cancer, systemic inflammation has an important role, and that CRP may be a useful indicator of inflammatory response. Results from various studies have proven that precancerous lesions are derived from systemic inflammation and local inflammation of the mucous membrane as part of the process of cell degeneration, and that colorectal cancer progresses from adenoma to adenocarcinoma [17,18].

Therefore, the objective of the current investigation is to assess the prognostic significance of preoperative serum VEGF, IL-6, and CRP levels as indicators of outcome in patients with colorectal cancer.

## Methods

### Patients

A total of 143 patients who underwent surgery for colorectal cancer at Dong-A University Hospital between December 2005 and December 2008 were enrolled in this study. All patients had histologically confirmed adenocarcinoma of the colon or rectum, and a 132 of the 143 had undergone a potentially curative resection. A 11 of the 143 patients showed microscopic evidence of residual disease; therefore, they were excluded. The control group consisted of 50 persons who underwent health check-ups (25 men and 25 women; median age, 41.5 years; range, 18 to 68 years) absence of neoplastic disease was established in these patients through laboratory testing including serum VEGF, IL-6 and CRP levels. Staging was based on routine postoperative histopathological analysis and clinical assessment by the American Joint Committee on Cancer (AJCC) TNM staging system. Disease status of patients was evaluated by abdominal computed tomography after surgery. CT was checked every 3 months for the first 2 years, and then every 6 months for a total of 5 years. Colonoscopy was performed at 1 year after the resection. Clinical outcomes were followed from the date of surgery to either the date of death or July 2009. A total of 11 colorectal cancer patients died of malignancy and 6 patients were lost during the observation period. The median follow-up period was 18.53 months (range 0.73-43.17). The study was approved by the Institutional Review Board (IRB). All patients provided informed consent, and the hospital review board approved the study.

### Assay of serum VEGF, IL-6, and CRP

Venous blood sampling was conducted within a period of 7 days prior to surgery. Blood collected for VEGF and IL-6 serum level assessments was collected in plain tubes, and levels of serum VEGF and IL-6 were measured using commercially available enzyme-linked immunosorbent assays (ELISAs). Blood samples were centrifuged for 10 min at 3,000 r/min at -4°C. Serum was subsequently removed and stored at -80°C for use in biochemical analysis. Blood samples for CRP analysis were collected in serum separation tubes, and serum CRP levels were measured via immunoturbidimetry.

### Statistical analysis

Relationships and interactions of the prognostic factors were tested using the Mann-Whitney U-test and the Kruskal Wallis test. Serum levels of VEGF, IL-6, and CRP were expressed as means and median values. VEGF, IL-6, and CRP cut-off values for survival analysis were determined by ROC curve; 825 pg/mL, 11.68 pg/mL, and 0.185 mg/dL, respectively. Duration of recurrence of colorectal cancer and death measured from the date of surgery was referenced against disease free survival and overall survival time. Survival duration was calculated via the Kaplan-Meier method. The log-rank test was employed for comparison of cumulative survival rate and disease free survival in the patient group. For identification of independent factors significantly associated with patient prognosis, we used multivariate analyses employing the Cox proportional hazards model. A *p *value < 0.05 was considered statistically significant. Variables found to be significant at a level of *p *< 0.05 were considered eligible for multivatiate regression analysis. The Statistical Package for Social Sciences (SPSS) Version 14.0 (SPSS Inc, Chicago, IL, USA) for Windows was utilized for all statistical analyses.

## Results

Patients were classified according to their pathologic characteristics, included tumor size, depth of tumor invasion, status of lymph node (LN) metastasis, lymph node ratio, CEA, American Joint Committee on Cancer tumor-nodes-metastases (AJCC TNM) stage, sex, and age. Patients included 79 men and 53 women, with a median age of 62 years (range, 26-83 years). Characteristic data for the study population are shown in Table 1. Median follow-up duration was 19.25 months (range 0.73-43.17 months).

**Table 1:** Patient characteristics

	No. of patients	%
Total number of patients	132	
Sex		
Male	79	59.8
Female	53	40.2
Median age(range)	62(26-83)	
<60	52	39
≥60	80	61
CEA		
≤5 ng/mL	99	75.6
>5 ng/mL	32	24.4
Tumor size		
<5 cm	58	43.9
≥5 cm	74	56.1
Lymph node ratio		
pNr1 0.01-0.11	103	78
pNr2 0.12-0.24	17	12.9
pNr3 0.25-0.92	12	9.1
Primary Tumor		
pT1	12	9.1
pT2	19	14.4
pT3	90	68.2
pT4	11	8.3
Regional Lymph nodes		
N0	75	56.8
N1	36	27.3
N2	21	15.9
Differentiation		
Well	67	50.8
Moderate	52	39.4
Poor	5	3.8
Mucinous	8	6.1
TNM stage		
Stage I	27	20.5
StageII	48	36.4
StageIII	57	43.2

CEA: carcinoembryonic antigen, normal range 0-5 ng/mL

Mean and median levels of VEGF and CRP in the colorectal cancer group were significantly higher than in the normal control group; 620 vs. 334 pg/mL and 541 vs. 312 pg/mL (*p *< 0.001); 1.12 vs. 0.43 mg/dL and 0.25 vs. 0.07 mg/dL (*p *= 0.001). However mean and median level of IL-6 in patients were not significantly higher than in the control group; 13.77 vs. 5.65 pg/mL and 5.74 vs. 5.30 pg/mL (*p *= 0.437), respectively (Table 2).

**Table 2:** The mean and median levels of VEGF, IL-6 and CRP in colorectal cancer patients and control group

	Patients (n = 132)		Control (n = 50)		P-value
		
	Mean	Median (range)	Mean	Median (range)	
VEGF	608	528 (122-3242)	334	312 (16-1121)	< 0.001
IL-6	14.33	6.00 (1.02-139.17)	5.65	5.30 (4.50-13.78)	0.327
CRP	1.05	0.22 (0.00-18.40)	0.43	0.07 (0.02-6.94)	0.002

** Range of Values (Serum); **VEGF **62-707 pg/mL; **IL-6 **3.12-15.5 pg/mL; **CRP **0-0.5 mg/dL

Relationships between VEGF, IL-6, and CRP levels, and clinicopathologic variables were tested using the Mann-Whitney U-test and the Kruskal test. Details of these relations are listed in Table 3. VEGF was found to be significantly correlated with tumor size (*p *= 0.012) and CEA (*p *= 0.038). But no significantly correlations were observed between VEGF and other variables. IL-6 and CRP levels were not correlated with other pathological findings.

**Table 3:** Correlation between the IL-6, VEGF, CRP, and clinicopathological parameters

	VEGF pg/ml			IL-6 pg/ml			CRP mg/dL		
	**Mean**	**Median (range)**	***p***	**Mean**	**Median (range)**	***p***	**Mean**	**Median (range)**	***p***

Sex									
Male	656	567 (131-3242)	0048	13.12	5.03 (1.02-139.17)	0.019	1.24	0.25 (0.01-18.40)	0.827
Female	567	469 (123-2001)		16.14	7.71 (1.25-90.06)		0.83	0.16 (0.00-8.41)	

Age									
< 60	634	569 (123-1620)	0.363	15.31	5.61 (1.02-90.06)	0.854	1.15	0.17 (0.02-8.41)	0.610
≥ 60	593	494 (131-3242)		13.70	6.63 (1.58-139.17)		0.98	0.24 (0.00-18.40)	

CEA									
≤ 5	583	493 (123-32.42)	0.038	12.98	5.70 (1.02-94.11)	0.255	1.14	0.20 (0.01-18.40)	0.824
> 5	680	635 (131-1619)		18.91	8.0 (1.58-139.17)		0.80	0.25 (0.02-6.97)	

Primary tumor									
pT1	452	422 (123-892)	0.540	18.60	3.93 (2.18-94.11)	0.549	1.02	0.16 (0.01-6.22)	0.905
pT2	565	471 (139-1585)		14.78	7.74 (1.02-70.96)		0.67	0.33 (0.02-3.43)	
pT3	639	546 (126-3242)		13.35	6.02 (1.25-139.17)		1.17	0.24 (0.00-18.40)	
pT4	606	567 (174-1216)		17.00	6.05 (3.44-88.96)		0.77	0.53 (0.03-2.29)	

Regional Lymph nodes									
N0	652	539 (123-3242)	0.557	13.32	6.49 (1.02-94.11)	0.533	1.04	0.26 (0.01-10.09)	0.432
N1	579	562 (139-1098)		8.53	5.66 (1.49-30.02)		1.23	0.13 (0.00-18.40	
N2	560	432 (156-1620)		27.92	6.31 (1.25-139.17)		0.79	0.20 (0.02-7.83)	

Lymph node ratio									

pNr1 0.01-0.11	634	558 (123-3242)	0.215	11.89	5.58 (1.02-94.11)	0.242	1.16	0.25 (0.0-18.40)	0.352
pNr2 0.12-0.24	495	495 (145-1619)		20.86	6.31 (1.91-90.06)		0.76	0.11 (0.00-6.97)	
pNr3 0.25-0.92	446	430 (156-1068)		26.10	10.15 (1.25-139.17)		0.54	0.17 (0.04-2.18)	

Size									
< 5 cm	550	463 (123-3242)	0.012	10.58	5.15 (1.02-76.79)	0.129	1.32	1.15 (0.00-18.40)	0.739
≥ 5 cm	655	577 (126-2001)		12.28	7.15 (1.25-139.17)		0.84	0.25 (0.01-8.41)	

Stage									
Stage I	547	471 (123-1586)	0.320	17.60	5.52 (1.02-94.11)	0.907	0.88	0.16 (0.01-6.22)	0.681
Stage II	711	573 (126-3242)		10.94	10.94 (1.58-93.08)		1.13	0.28 (0.01-10.09)	
Stage III	553	495 (139-1620)		15.67	5.97 (1.25-139.47)		1.07	0.13 (0.00-18.40)	

Differentiation									
Well	644	495 (123-3242)	0.234	12.07	5.49 (1.02-94.11)	0.275	0.75	0.25 (0.01-6.97)	0.957
Moderate	541	511 (133-1620)		18.07	7.45 (1.49-139.17)		1.60	0.15 (0.00-18.40)	
Poor	531	520 (174-1083)		7.45	8.42 (2.65-11.88)		0.28	0.20 (0.00-0.54)	
Mucinous	805	663 (448-1258)		13.35	8.30 (1.25-39.03)		0.52	0.18 (0.02-1.60)	

**Figure 1:**
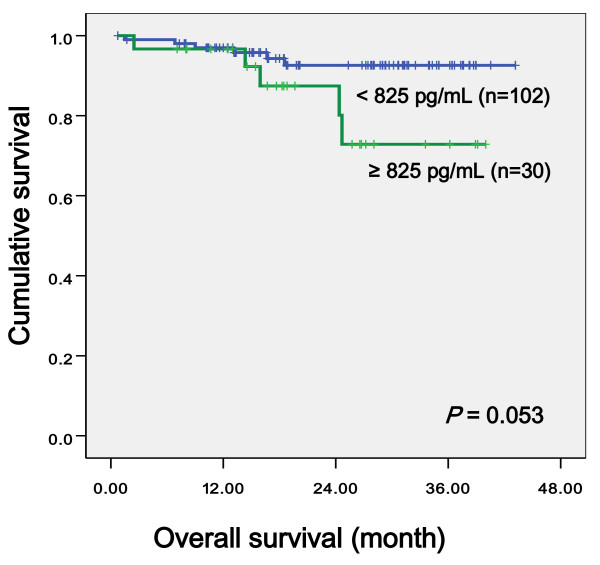
**Overall survival curve according to VEGF level**.

**Figure 2:**
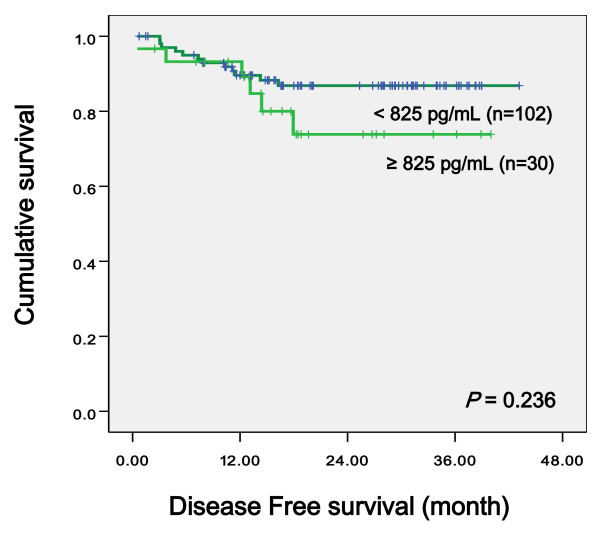
**Disease free survival curve according to VEGF level**.

The relationship between serum levels of VEGF, IL-6, CRP, and clinicopahologic variables was evaluated using the Kaplan-Meier method. We established the cutoff value for VEGF (825 pg/mL), IL-6 (8.09 pg/mL), and CRP (0.51 mg/dL) by ROC curve. Univariate log-rank analysis showed that CEA (*p *< 0.001), regional lymph nodes status (*p *< 0.001), tumor size (*p *= 0.029), and lymph node ratio (*p *= 0.001) were reduced overall survival. We noted that high VEGF levels tended to reduce overall survival (*p *= 0.053, Figure 1), but not significantly. VEGF levels demonstrated no significance with regard to disease free survival (*p *= 0.236, Figure 2). IL-6 and CRP levels provided no significant evidence with regard to overall patient survival (*p *= 0.956, *p *= 0.607 respectively) and disease free survival (*p *= 0.531, *p *= 0.701, respectively). Multivariate regression analysis using Cox's proportional hazards model revealed that VEGF (*p *= 0.032, HR = 4.779), CEA (*p *= 0.012, HR = 7.981), regional lymph nodes status (*p *< 0.001), and age (p = 0.005, HR 11.91) were independent prognostic factors for survival of colorectal cancer (Table 4).

**Table 4:** Multivariate analysis on OS and DFS in 143 colorectal cancer patients

	DFS			OS		
		
	HR	95% C.I	***p***	HR	95% C.I	***p***
VEGF, <825 vs. ≥825 pg/mL*	1.281	0.41-4.03	0.672	4.779	1.15-19.94	0.032
IL-6, <11.68 vs. ≥11.68 pg/mL*	1.284	0.44-3.73	0.646	1.391	0.36-5.44	0.634
CRP, <0.18 vs. ≥0.18 mg/dL*	1.030	0.38-2.80	0.954	0.869	0.22-3.51	0.844
CEA, <5 vs. ≥5 ng/mL	8.391	2.51-28.07	0.001	7.981	1.44-44.10	0.012
Tumor size, <5 vs. ≥5 cm	0.210	0.06-0.81	0.023	3.448	0.29-41.06	0.327
T, T1 vs. T2 vs. T3 vs. T4	2.479	0.94-6.57	0.068	5.963	0.76-46.87	0.090
N, N0 vs. N1 vs. N2	4.807	2.38-9.72	<0.001	5.867	2.86-86.20	0.002
Stage, I vs. II vs. III	0.783	0.12-5.10	0.798	0.042	0.00-0.67	0.025
Age, <60 vs. ≥60	0.726	0.20-2.59	0.622	11.91	2.14-66.33	0.005

* cutoff value by ROC curve

## Discussion

Recent studies have shown the correlation between tissue VEGF expression and tumor aggressiveness in colon carcinoma. VEGF expression was found to be higher in patients who had metastatic tumors compared with patients who had non-metastatic tumors [10], [19,20]. Takahashi et al [21] showed that VEGF expression level in patients with lymph node negative colon carcinoma was significantly associated with time of recurrence, whereas Cascinu et al [22] confirmed that positive VEGF status was associated with a significant reduction in 5-year DFS rate.

The prognostic impact of serum VEGF levels in cancer patients has been evaluated in a few studies. A large study by Danish Colorectal Cancer Study Group found that high preoperative VEFG concentrations were associated with reduced overall survival with respect to patients with colon carcinoma who had lower VEGF serum values, and suggested a biologically relevant role for serum VEGF concentration in patients with colorectal carcinoma [23-25].

According to findings from recent studies, VEGF has been proposed as a potential biomarker for prediction of colorectal cancer prognosis. De Vita et al [26] determined preoperative and post operative serum VEGF levels by ELISA in patients with colon cancer who underwent surgery. They found that preoperative VEGF was significantly higher in the colon cancer compared with the control group, and preoperative VEGF was significantly lower in patients who underwent curative surgery compared with patients who underwent non-curative surgery. Therefore, they suggested that preoperative serum VEGF levels might be useful for prediction of outcome in patients with colon cancer who underwent surgery. Another study investigated VEGF expression in colorectal cancer specimens by performance of immunohistochemistry on tissue microarrays [27]. In that study, the authors found VEGF expression in all tumors evaluated; they also found greater intensity of VEGF staining, greater lymph node metastasis, higher stage, and poorer disease-specific survival. They concluded that VEGF expression in colorectal cancer appears to be an independent prognostic marker of tumor behavior, and that it may be useful in identification of patients with unfavorable clinical outcome.

In our study, preoperative serum VEGF level by ELISA in the patient group was significantly higher than levels in the control group (*p *< 0.001) and preoperative serum VEGF levels were significantly correlated with CEA levels (*p *= 0.038) and tumor size (*p *= 0.012). When we established the cutoff value for VEGF (825 pg/mL) by ROC curve, we noted that high VEGF levels tended to reduce overall survival, but was not significantly (*p *= 0.053). No significant correlation was found between VEGF preoperative levels and disease stage, gender, tumor depth, and status of lymph node metastasis.

Currently, ample literature is available with regard to the role of various cytokines in colorectal cancer patients. Mechanisms leading to IL-6 induction and to IL-6 presence in high concentrations in the serum of those patients include CEA-induced IL-6 production by Kupffer cells, malignancy-related chronic stress leading to increased IL-6 blood concentrations, as well as direct IL-6 production and secretion by tumor-associated macrophages or the tumor cells themselves. According to some studies that evaluated clinical significance of IL-6 pre-treatment levels, IL-6 concentrations reflected disease status, and were commonly associated with metastatic disease [28,29]. Findings from other research did not reveal a significant association between IL-6 and tumor stage, or other clinical findings [30]. However, the authors of that study found significant correlation between large tumor size and hepatic metastasis correlated with elevated IL-6 levels significantly. Furthermore, high IL-6 levels were associated with reduced overall survival. Different from prior studies, was the fact that it was comprised more of non-metastatic stages, which strengthens the clinical relevance of this observation. They suggested that in the absence of advanced metastatic disease, IL-6 levels may be used as an adverse prognostic marker.

CRP is synthesized in hepatocytes and produced by other organs in response to release of IL-6 by monocytes and other immune cells [31]. Increased CRP level has been previously reported in patients with impaired T lymphocyte response; thus this mechanism is considered to have an association with the immune system and poor survival rate [32]. Elevated levels of this marker of infection have been associated with increased risk of cardiovascular disease [33], and have been identified as a poor prognostic factor for several diseases, including a variety of cancers in a number of previous studies [34-37]. Findings from studies in patients with colorectal cancer have indicated that those with elevated serum CRP concentrations had poorer prognoses than those whose CRP level did not increase. In addition, it has been suggested that increased CRP was associated with more frequent local tumor invasion [38], more advanced pathologic stage [39], a higher rate of recurrence [40], and reduced overall survival [30].

Unlike results from earlier studies, we were unable to determine the correlation between IL-6 and CRP and prognostic factors. Although IL-6 and CRP levels showed no correlation with other pathological findings, mean and median levels of CRP in colorectal cancer patients were significantly higher than those in normal individuals (*p *= 0.001). When we established the cutoff value for IL-6 (11.68 pg/mL), and CRP (0.185 mg/dL) by ROC curve, IL-6 and CRP showed no significant evidence with regard to disease free survival (*p *= 0.701, *p *= 0.531, respectively) and overall survival (*p *= 0.563, *p *= 0.572, respectively).

This retrospective study had some limitations. First, the patient group was restricted to patients who underwent surgery. Thus this study included more patients who were able to undergo surgery at a lower stage (not included stage IV). And we did not consider patients who underwent surgery or those who had any concomitant disease suspected of raising VEGF, IL-6, or CRP (i.e., chronic inflammatory disorders, diabetes mellitus, ischemic heart disease, etc.). Thus, these were possible causes for misinterpretation of results. The most important limitation of this study is the lack of post operative VEGF, IL-6 and CRP levels. De Vita et al.[26] suggested preoperative VEGF and CEA levels as good prognostic indicators for curative and noncurative surgery, and VEGF levels dropped significantly after surgery, with a further downward trend until the 30th postoperative day. However if we had determined postoperative serum levels of VEGF, IL-6, and CRP, we would have been able to obtain more information with regard to prognosis. For examples, normalization of postoperative serum levels could suggest radical surgery; serum level decrement would show secretion of the marker by the tumor, and patients undergoing potentially curative surgery who does not show serum level normalization of the marker should undergo treatment with chemotherapy regardless of the TNM stage, and increased serum levels during the follow-up period could be prediction of recurrence.

Conclusively, high preoperative VEGF levels were associated with tumor size, higher CEA, and VEGF was independent of overall survival with CEA and lymph node status. Thus we surmise that the prognosis in colorectal cancer patients with high VEGF levels would be generally poor.

## Conclusions

As a result of the current study, VEGF is suggested as an important prognostic factor for colorectal cancer. For the future, more prospective studies will be necessary for investigation of predictive biomarkers for colorectal cancer.

## Competing interests

The authors declare that they have no competing interests.

## Authors' contributions

KAK performed the statistical analysis and drafted the manuscript. H-CK collected the data, performed the statistical analysis with interpretation and critically revised the manuscript. SHK, SYO and SL performed the chemotherapy for patients and revised the manuscript. HJC and KJP performed the operations for patients and revised the manuscript. KHK, J-YH, RYG and MSR carried out the immunoassays. JHL and H-JK conceived of the study, and approved the final manuscript. All authors read and approved the final manuscript.

## Pre-publication history

The pre-publication history for this paper can be accessed here:

http://www.biomedcentral.com/1471-2407/10/203/prepub
